# Multiple Strategies Confirm the Anti Hepatocellular Carcinoma Effect of Cinnamic Acid Based on the PI3k-AKT Pathway

**DOI:** 10.3390/ph18081205

**Published:** 2025-08-14

**Authors:** Jiageng Guo, Lijiao Yan, Qi Yang, Huaying Li, Yu Tian, Jieyi Yang, Jinling Xie, Fan Zhang, Erwei Hao

**Affiliations:** 1 Guangxi Key Laboratory of TCM Formulas Theory and Transformation for Damp Diseases, Guangxi University of Chinese Medicine, Nanning 530000, China; 2 Guangxi Key Laboratory of Efficacy Study on Chinese Materia Medica, Guangxi University of Chinese Medicine, Nanning 530000, China; 3 University Engineering Research Center of Reutilization of Traditional Chinese Medicine Resources, Nanning 530000, China; 4 Engineering Research Center of Innovative Drugs for Traditional Chinese Medicine and Zhuang & Yao Medicine, Ministry of Education, Guangxi University of Chinese Medicine, Nanning 530000, China

**Keywords:** hepatocellular carcinoma, cinnamic acid, network pharmacology, molecular docking, PI3K/AKT signaling pathway, cell apoptosis

## Abstract

**Background:** Hepatocellular carcinoma is one of the leading causes of cancer-related deaths worldwide. Its high recurrence rate and limited treatment options underscore the urgent need for the development of new and highly effective drugs. **Methods**: This study systematically explores the molecular mechanism of cinnamic acid against hepatocellular carcinoma through integrated machine learning prediction, network pharmacological analysis and in vitro experimental verification. **Results**: The prediction of anti-tumor activity based on the random forest model showed that cinnamic acid has significant anti-tumor potential (probability = 0.69). Network pharmacology screened 185 intersection targets of cinnamic acid and liver cancer, of which 39 core targets (such as PIK3R1, AKT1, MAPK1) were identified as key regulatory hubs through protein interaction network and topological analysis. Functional enrichment analysis showed that these targets were mainly enriched in the PI3K/AKT signaling pathway (*p* = 2.1 × 10^−12^), the cancer pathway (*p* = 3.8 × 10^−10^), and apoptosis-related biological processes. Molecular docking validation revealed that the binding energies of cinnamic acid with the 19 core targets were all below −5 kcal/mol, a threshold indicating strong binding affinity in molecular docking. The binding modes to PIK3R1 (−5.4 kcal/mol) and AKT1 (−5.1 kcal/mol) stabilized through hydrogen bonding. In vitro, cinnamic acid dose-dependently inhibited Hep3B proliferation/migration, induced apoptosis, downregulated PI3K, p-AKT, and Bcl-2, and upregulated Bax and Caspase-3/8. **Conclusions**: This study systematically reveals, for the first time, that the multi-target mechanism of cinnamic acid exerts anti-hepatic cancer effects by targeting the PI3K/AKT signaling pathway, supporting its potential as a natural anti-tumor drug.

## 1. Background

Hepatocellular carcinoma (HCC) is a highly aggressive malignant tumor originating from hepatocytes, with clinical characteristics such as poor prognosis and high recurrence rate [1]. Due to the concealment of early symptoms, more than 60% of patients have lost the chance of radical treatment such as liver resection or liver transplantation when diagnosed [2]. Even if they undergo radical resection, patients still face a 50% 3-year recurrence rate and 70% 5-year recurrence rate [3,4]. The treatment methods for advanced HCC are limited. Although the initial response rate of chemotherapy drugs represented by doxorubicin is 79%, the long-term clinical response rate is less than 20%, and it has failed to significantly improve the overall survival of patients (*p* > 0.05) [5]. Epidemiological data show that the 5-year survival rate of HCC patients in China is only 12% [5], and the development of highly efficient and less toxic targeted therapeutic drugs has become an urgent need for current research.

In recent years, the convergence of bioinformatics and in silico prediction has reshaped the landscape of drug discovery [6]. Machine learning models rapidly mine large pharmacological datasets to prioritize bioactive molecules [7]. Molecular docking then predicts ligand–target binding modes with atomic detail, and molecular dynamics simulations track the ensuing conformational changes over time [6,7,8]. When used in combination, these tools shorten development timelines and offer an effective route to dissect the multi-target, anti-tumor mechanisms of natural products.

Cinnamon, a dried bark originating from Cinnamomum cassia Presl (Camphoraceous), also referred to as fungus cinnamon and peony cinnamon, primarily originates from Guangxi, Guangdong, and Hainan provinces in China [9]. In the realm of traditional medicine, cinnamon aids in the regulation of the body’s Yang energy by limiting inflammation, promoting the return of energy to its source, dispersing coldness, alleviating discomfort, and promoting the flow of energy in the body’s meridians [10]. Recent studies in pharmacology indicate that cinnamic acid, which is the primary active component in cinnamon, exhibits various biological activities, including anti-tumor effects, anti-inflammatory properties, antioxidant capabilities, and the regulation of metabolism [11,12,13,14,15,16]. Cinnamic acid has demonstrated significant tumor-suppressive effects on various cancers, including breast cancer, colon cancer, lung adenocarcinoma, and prostate cancer [17,18,19,20]. Despite these existing findings, the molecular mechanisms governing its anti-tumor actions—especially in the context of HCC—are not yet fully understood. Previous research has mostly focused on verifying its inhibitory effects in different tumor cell lines or investigating single pathways. However, HCC involves complex, multifactorial pathogenesis and drug resistance issues, requiring a more integrative investigation of cinnamic acid’s multi-target properties. Therefore, further exploration of cinnamic acid’s novel mechanisms in terms of HCC remains crucial for unlocking its full therapeutic potential.

The PI3K/AKT signaling pathway is the core hub for regulating tumor occurrence and development, and is involved in key biological processes such as cell proliferation, apoptosis, metabolic reprogramming and chemotherapy resistance [21]. In HCC, aberrant activation of the PI3K/AKT pathway frequently correlates with tumor progression, metastasis, and poor prognosis [22]. This signaling pathway not only promotes malignant behaviors like uncontrolled cell cycle progression and anti-apoptotic signaling, but also contributes to angiogenesis and drug resistance [23]. The research and development of targeted inhibitors against this pathway has become an important field in anti-tumor treatment, especially for HCC patients who often exhibit high AKT phosphorylation levels [24].

The research and development of targeted inhibitors against this pathway has become a major focus in anti-tumor therapy. Based on this, this study intends to use interdisciplinary strategies to (1) construct a machine learning model to predict the anti-tumor activity of cinnamic acid, (2) use bioinformatics technology to screen its potential targets, (3) use molecular docking and dynamics simulation and analysis of the details of ligand-target interactions, and, in combination with in vitro experiments, (4) use cinnamic acid to induce apoptosis on Hep3B hepatocarcinoma cells and determine the regulatory effect of the PI3K/AKT pathway. The first systematic study has confirmed that cinnamic acid exhibits significant anti-tumor properties, primarily through the regulation of the PI3K/AKT signaling pathway. Experimental results demonstrate that this compound effectively inhibits the growth and metastasis of Hep3B cells while promoting apoptosis, thereby suppressing liver cancer. This discovery not only establishes a theoretical foundation for the development of anti-tumor drugs derived from natural products but also offers new perspectives and paradigms for contemporary natural product research.

## 2. Results

### 2.1. Performance of Different Anti-Tumor Prediction Models

**Figure 1:**
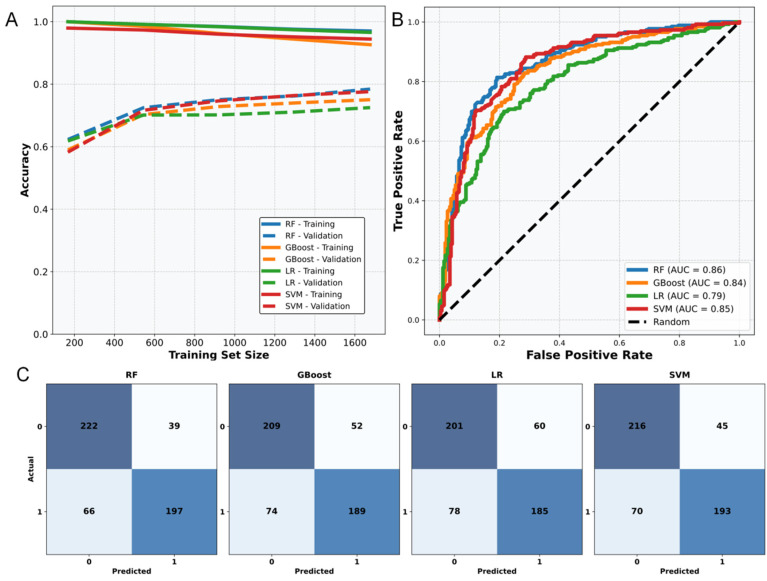
(**A**,**B**) represent the learning curves and ROC curves of four learning algorithms, with different colored lines representing distinct learning models. In (**A**), the dashed lines correspond to the prediction results on the test set. The absence of intersection between the solid and dashed lines in (**A**) indicates that no overfitting has occurred. In (**B**), the closer the curve is to the top-left corner of the coordinates, the stronger its generalization ability. (**C**) is the confusion matrix of the four learning models, and it can be simply interpreted that the darker the colors in the top-left and bottom-right corners, the stronger the predictive performance for 0 (negative label) and 1 (positive label), respectively.

As shown in Table 1, four machine learning models, random forest (RF), gradient boost (GBoost), logistic regression (LR) and support vector machine (SVM) all showed reliable performance in anti-tumor activity prediction (AUC > 0.7). Among them, the RF model performed the best, with accuracy (0.80), F1 score (0.80), AUC (0.86) and sensitivity (0.76) significantly higher than other models (Figure 1A). ROC curve analysis further showed that the curve of the RF model is closest to the upper left corner of the coordinate system, indicating that it has the strongest ability to distinguish positive and negative samples (Figure 1A). Confusion matrix analysis showed that all models had slightly better predictive power for negative labels (non-anti-tumor activity) than positive labels (Figure 1B), which may be related to the higher diversity of negative samples in the dataset.

Curves of different colors represent different prediction models. The standard curve in the middle represents the model’s ability to not classify. The closer the curve is to the upper left corner of the coordinate system, the better its performance. B is the prediction confusion matrix of four models. The darker the color of the two, the stronger the prediction ability of this type of label. The upper left corner and the lower right corner represent the prediction results for 0 (negative label) and 1 (positive label), respectively.

### 2.2. Prediction of Anti-Tumor Activity of Cinnamic Acid and Verification Set

Based on the RF model’s prediction of 101 small molecules (including 100 random molecules and cinnamic acid), 68 molecules were determined to have potential anti-tumor activity (probability > 0.5). Among these, cinnamic acid ranks fourth with a probability of 0.69 (Table 2), which is significantly higher than the average probability of random molecules (0.52 ± 0.08), indicating that it has significant anti-tumor potential and deserves further experimental verification.

### 2.3. The Intersection Target Results of Cinnamic Acid and Liver Cancer

**Figure 2:**
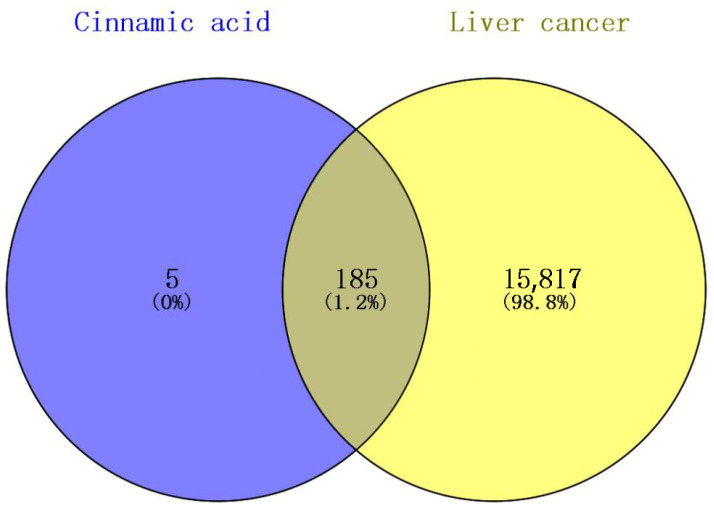
Venn diagram of cinnamic acid target and liver cancer target.

Through database mining, a total of 190 putative cinnamic acid targets and 16,003 liver cancer-related targets were collected. Venn analysis revealed that only 185 of these overlapped (Figure 2), representing approximately 1% of the overall HCC targets. Although this percentage may appear small, it likely highlights a focused subset of functionally relevant nodes specifically modulated by cinnamic acid. In other words, cinnamic acid does not act on the majority of liver cancer-associated targets, but rather on those more directly involved in critical pathways (e.g., PI3K/AKT), as subsequently supported by topological and enrichment analyses. Consequently, these intersection targets are presumed to be more crucial for driving tumor growth or progression and thus hold higher therapeutic potential for further investigation.

### 2.4. PPI Network Construction and Core Target Screening

**Figure 3:**
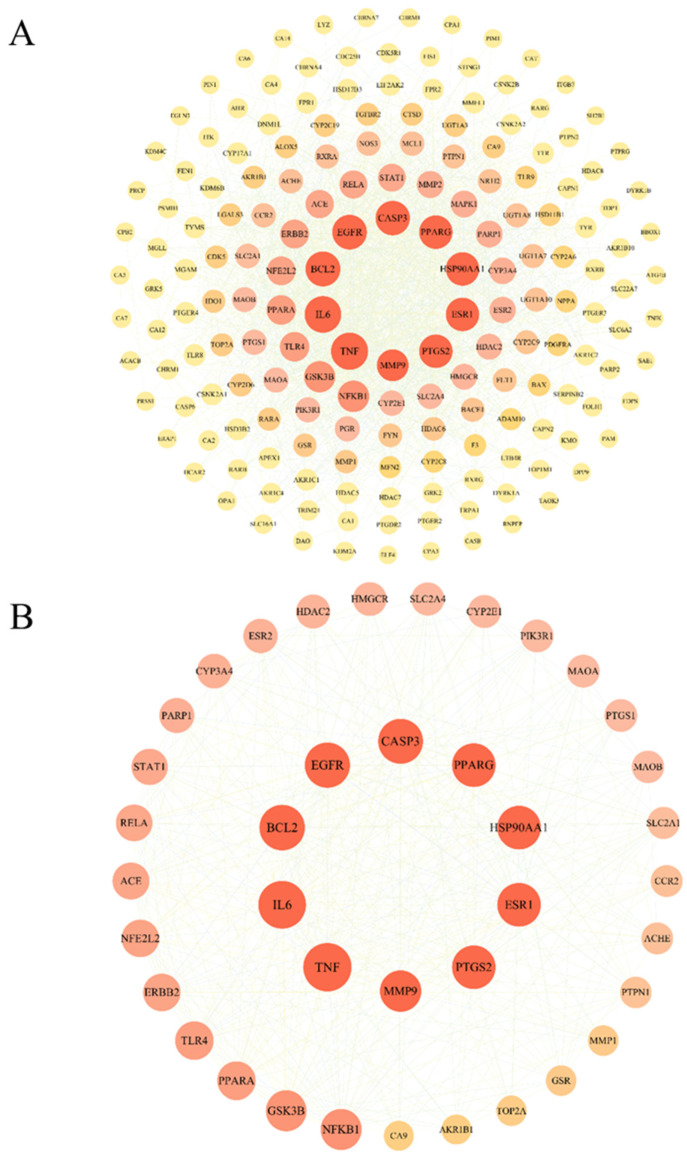
PPI and target screening results. (**A**) Protein–protein interaction (PPI) network and (**B**) core target network for cinnamic acid treatment of liver cancer.

The PPI network built by the STRING database contains 179 nodes and 1539 edges (confidence > 0.4) (Figure 3A). Through topological analysis (betweenness ≥ 257.821, closeness ≥ 0.00237, degree ≥ 17.195), 39 core targets were screened (Figure 3B), including PIK3R1, AKT1, MAPK1, etc., which may play a central role in the treatment of liver cancer.

### 2.5. GO Functional Enrichment Analysis and KEGG Pathway Enrichment Analysis

**Figure 4:**
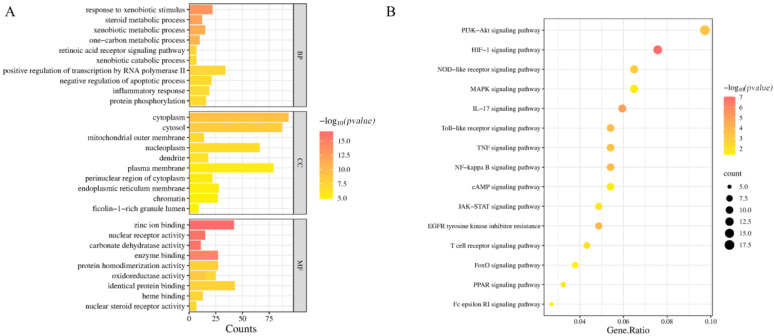
Enrichment analysis results. (**A**) Results of GO biological functional enrichment analysis and (**B**) results of KEGG pathway enrichment analysis.

GO analysis showed that intersection targets were significantly enriched in processes such as lipid metabolism regulation (BP), cell membrane compartment (CC), and kinase activity (MF) (Figure 4A). KEGG pathway analysis showed that PI3K-AKT signaling pathway (*p* = 2.1 × 10^−12^), cancer pathway (*p* = 3.8 × 10^−10^), and MAPK signaling pathway (*p* = 1.5 × 10^−8^) are the cores of the top three pathways (Figure 4B), suggesting that cinnamic acid may inhibit liver cancer progression by regulating these pathways.

### 2.6. Component-Pathway-Target-Disease Network Construction

**Figure 5:**
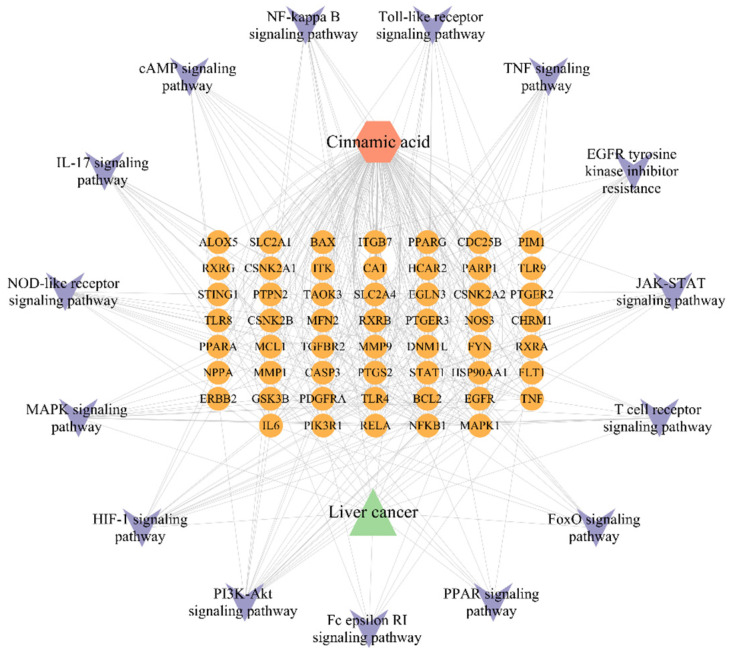
“Composition-Pathway-Target-Disease” network.

The network constructed by integrating the first 15 KEGG pathways and their associated 54 targets (Figure 5) shows that cinnamic acid acts on targets such as AKT1 and MAPK1 through the regulation of pathways such as PI3K-AKT, forming multi-target-multi-pathways. anti-hepatocellular carcinoma mechanism.

### 2.7. Verification of the Docking of Cinnamic Acid and PI3K-Akt Signaling Pathway-Related Target Proteins

**Figure 6:**
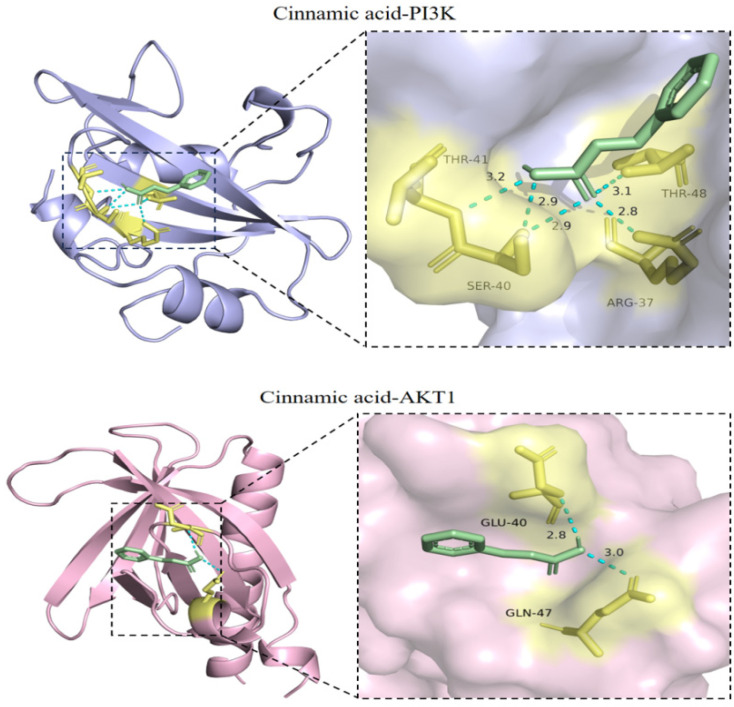
Visualization of cinnamic acid and PI3K and AKT1.

Using AutodockVina 1.5.7 software to calculate the binding energy of the docking of receptor proteins and ligand small molecules. The information related to receptors and binding energy is shown in Table 3. After visualizing the docking results (PI3K and AKT1) with the help of Pymol software, the amino acid binding in each conformation is obtained. The distance between the site and hydrogen bond and the visualization of molecular docking is shown in Figure 6.

From the docking results, it can be seen that the binding energy of cinnamic acid after docking with 19 proteins is −5 kcal/mol, indicating that it has a good docking effect, its docking activity is good, and its conformation is relatively stable. From the visualization, it can be seen that cinnamic acid is in the protein PIK3R1 (binding energy). The interacting amino acid sites on −5.4 kcal/mol are ARG37, SER40, and THR41, and the hydrogen bond distances are 2.1, 2.8, 2.9, 2.9, and 3.2, respectively. Cinnamic acid is on the protein AKT1 (binding energy −5.1 kcal/mol). The interacting amino acid sites are GLU40 and GLN47, and the hydrogen bond distances are 2.8 and 3.0 respectively.

### 2.8. Cell Viability Assay

**Figure 7:**
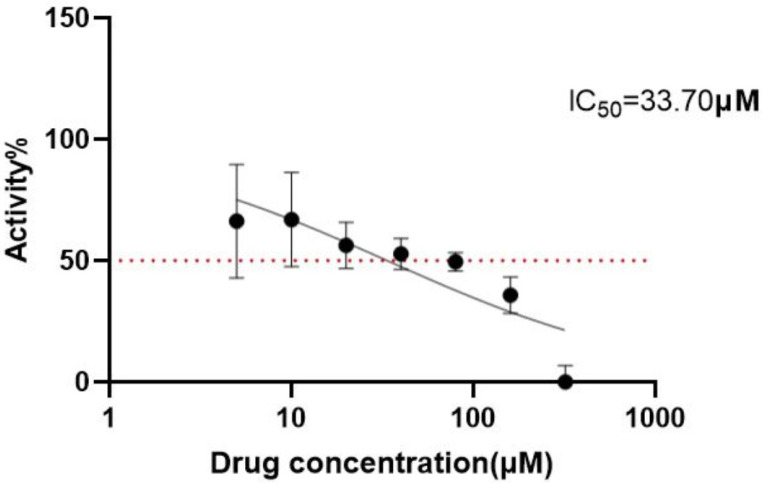
IC50 curve of cinnamic acid against Hep3B cells.

The inhibitory effect of cinnamic acid on Hep3B cell proliferation was evaluated by CCK8 experiments, and Hep3B cells were seeded at a density of 6000 cells per well in 96-well plates. The results show that, as drug concentration (5–320 μM) increased, cell survival showed a dose-dependent decrease (Figure 7). Calculated by nonlinear regression analysis, the half-inhibiting concentration (IC_50_) of cinnamic acid on Hep3B cells was 33.70 μM (95% CI: 22.52–49.98 μM), indicating that it has significant anti-hepatocellular proliferation activity.

### 2.9. Observation of Morphological Changes

**Figure 8:**
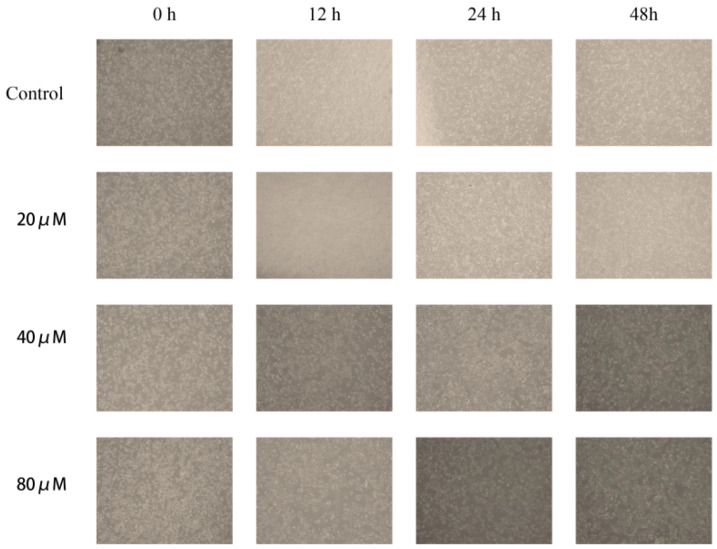
Effect of cinnamic acid on the morphology of Hep3B cells. Images were taken under an inverted microscope at 200× magnification. The scale bar represents 500 µm.

Hep3B cells were seeded in 6-well plates at a density of 3 × 10^5^ cells per well. Observed under an inverted microscope, we found that the control group Hep3B cells adhered well and had regular morphology and tight cell junctions (Figure 8). After 48 h of treatment with 20–80 μM cinnamic acid, the cell density significantly decreased. The cells gradually adopted a rounded morphology, the cell space increased, and apoptotic characteristics such as membrane blebbing and cell shrinkage became evident (Figure 8). In the 80 μM treatment group, the number of live cells decreased significantly, and the cell fragments increased. Although these changes are consistent with apoptosis-like morphology, morphological observation alone is insufficient to confirm programmed cell death. Therefore, additional evidence—such as flow cytometry-based Annexin V/PI staining and apoptotic protein measurements—was employed to verify that cinnamic acid indeed induces apoptosis in Hep3B cells. The results here preliminarily support our conclusion that higher concentrations of cinnamic acid induce apoptotic cell death.

### 2.10. Cinnamic Acid Inhibits Hep3B Cell Migration

**Figure 9:**
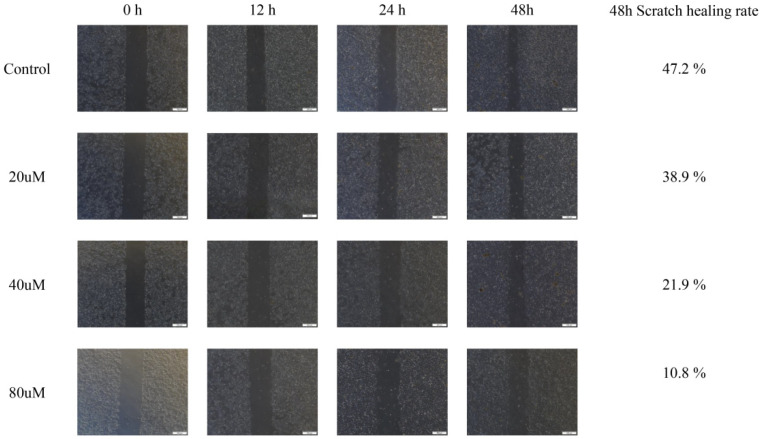
Representative images of scratch healing and statistics on scratch healing rate. Images were taken under an inverted microscope at 200× magnification. The scale bar represents 500 µm.

The results of the scratch experiment show that cinnamic acid significantly inhibited the migration ability of Hep3B cells (Figure 9). The healing rate of scratch at 48 h in the control group was 47.2%, while the healing rates in the 20 μM, 40 μM and 80 μM treatment groups decreased to 38.9%, 21.9%, and 10.8%, respectively (Figure 9). Further analysis showed that the migration inhibitory effect of cinnamic acid was dose-dependent (R^2^ = 0.93), suggesting that it may inhibit liver cancer metastasis by interfering with cytoskeleton recombination or matrix adhesion pathway.

Although no formal statistical test was conducted due to single-sample measurements in this preliminary experiment, the data indicate a clear trend of reduced migratory capability with increasing cinnamic acid concentrations.

### 2.11. Cinnamic Acid Promotes Apoptosis of Hep3B Cells

**Figure 10:**
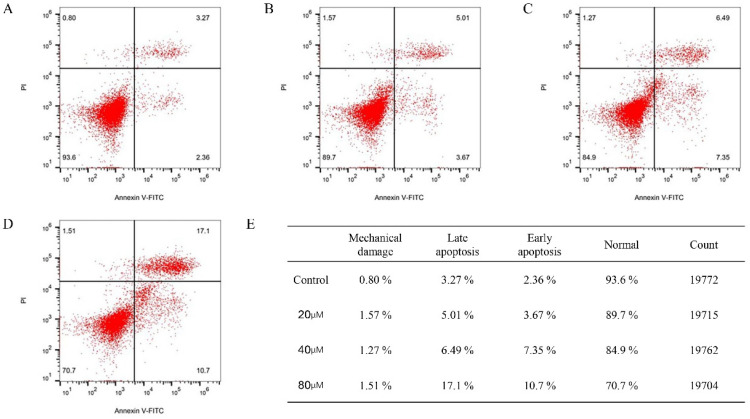
Flow cytometer analysis results and result statistics. (**A**) Control group: majority of cells remain in the normal quadrant (93.6%), with low percentages of mechanical damage (0.80%), late apoptosis (3.27%), and early apoptosis (2.36%). (**B**) 20 μM cinnamic acid treatment: slight increase in late apoptosis (5.01%) and early apoptosis (3.67%) compared with control, accompanied by a small reduction in normal cells (89.7%). (**C**) 40 μM cinnamic acid treatment: further elevation in late apoptosis (6.49%) and early apoptosis (7.35%), with a decline in normal cells to 84.9%. (**D**) 80 μM cinnamic acid treatment: marked increase in late apoptosis (17.1%) and early apoptosis (10.7%), with a pronounced reduction in normal cells to 70.7%. (**E**) Statistical summary of cell percentages in each quadrant (mechanical damage, late apoptosis, early apoptosis, and normal) for all groups, with total event counts listed.

Flow cytometry results showed that cinnamic acid treatment significantly increased the apoptosis rate of Hep3B cells in a dose-dependent manner (Figure 10). In the 80 μM treatment group, the total apoptosis rate (early + late) reached 27.8%, significantly higher than that of the control group (5.63%). Among them, the proportion of late apoptotic cells increased from 3.27% in the control group to 17.1%, the proportion of early apoptotic cells increased from 2.36% to 10.7%, and the proportion of normal cells decreased from 93.6% to 70.7% (Figure 10E).

### 2.12. Cinnamic Acid Regulates PI3K/AKT Signaling Pathway

**Figure 11:**
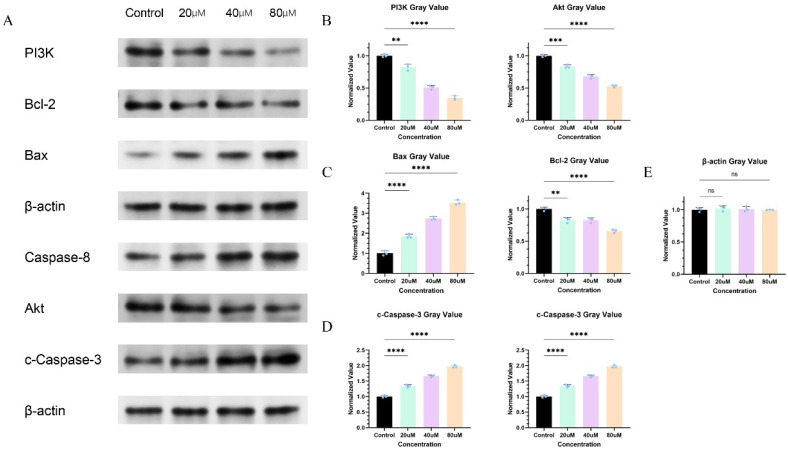
Western blot protein band results and quantitative gray value analysis. (**A**) Representative Western blot bands of PI3K, Akt, Bcl-2, Bax, Caspase-8, and cleaved-Caspase-3 in Hep3B cells treated with different concentrations of cinnamic acid (20, 40, and 80 μM) compared with the control group; β-actin was used as a loading control. (**B**) Quantitative gray value analysis of PI3K and Akt protein expression levels showing a concentration-dependent decrease after cinnamic acid treatment. (**C**) Quantitative gray value analysis of Bax and Bcl-2 protein expression levels, showing increased Bax and decreased Bcl-2 levels with higher cinnamic acid concentrations. (**D**) Quantitative gray value analysis of Caspase-8 and cleaved-Caspase-3 protein expression levels, showing significant upregulation in treated groups. (**E**) Quantitative gray value analysis of β-actin showing no significant difference among groups. Statistical significance: ns, not significant; ** *p* < 0.01; *** *p* < 0.001; **** *p* < 0.0001.

Western blot analysis showed that cinnamic acid treatment significantly downregulated protein expression levels of PI3K and AKT in Hep3B cells (Figure 11A). The PI3K expression in the 80 μM treatment group was 67.9% lower than that in the control group (Figure 11B–E), and the AKT expression was 45.9% lower than that in the control group, indicating that cinnamic acid inhibited AKT synthesis. This result is consistent with the high affinity binding of cinnamic acid to AKT1 in the PI3K/AKT pathway enrichment predicted by network pharmacology (Figure 4) and molecular docking (binding energy: −5.1 kcal/mol, Figure 6). We also tested the expressions of Bcl-2, Bax, Caspase-8 and c-Caspace-3 related to the classic apoptosis pathway, finding that the expressions of Bcl-2 in the 80 μM treatment group were 35.1% lower than those in the control group, and that the expressions of Bax protein were found in the 80 μM treatment group. However, we acknowledge that the phosphorylation status of AKT is a critical indicator of its activation. Due to experimental constraints, we did not assess p-AKT in this study, which remains a limitation. Future work will incorporate p-AKT/AKT ratio analyses to more precisely evaluate the impact of cinnamic acid on AKT activity. Such expanded data could more accurately delineate the role of cinnamic acid in modulating this pivotal pathway and further substantiate its therapeutic potential against HCC. The expression of Caspase-8 and c-Caspace-3 was increased by 282.4%, and the above results suggest that cinnamic acid induces apoptosis signaling by blocking PI3K/AKT signaling and exerting an anti-hepatic cancer effect. These findings reflect the well-established crosstalk between the PI3K/AKT pathway and the mitochondrial apoptosis pathway. Specifically, active AKT can enhance cell survival by maintaining elevated Bcl-2 levels and suppressing Bax-dependent mitochondrial membrane permeabilization. When cinnamic acid inhibits PI3K/AKT signaling, this survival advantage is diminished, thereby shifting the balance in favor of apoptotic signals. Consequently, the increased Bax/Bcl-2 ratio and activation of Caspase-3/8 contribute to the execution of apoptosis in Hep3B cells. Taken together, the suppression of PI3K/AKT and the concurrent induction of apoptosis-related proteins underscore cinnamic acid’s potential to disrupt multiple aspects of hepatocellular carcinoma cell survival.

### 2.13. Molecular Dynamics Simulation Corroborates the Binding Stability of Cinnamic Acid to AKT1 and PIK3R1

**Figure 12:**
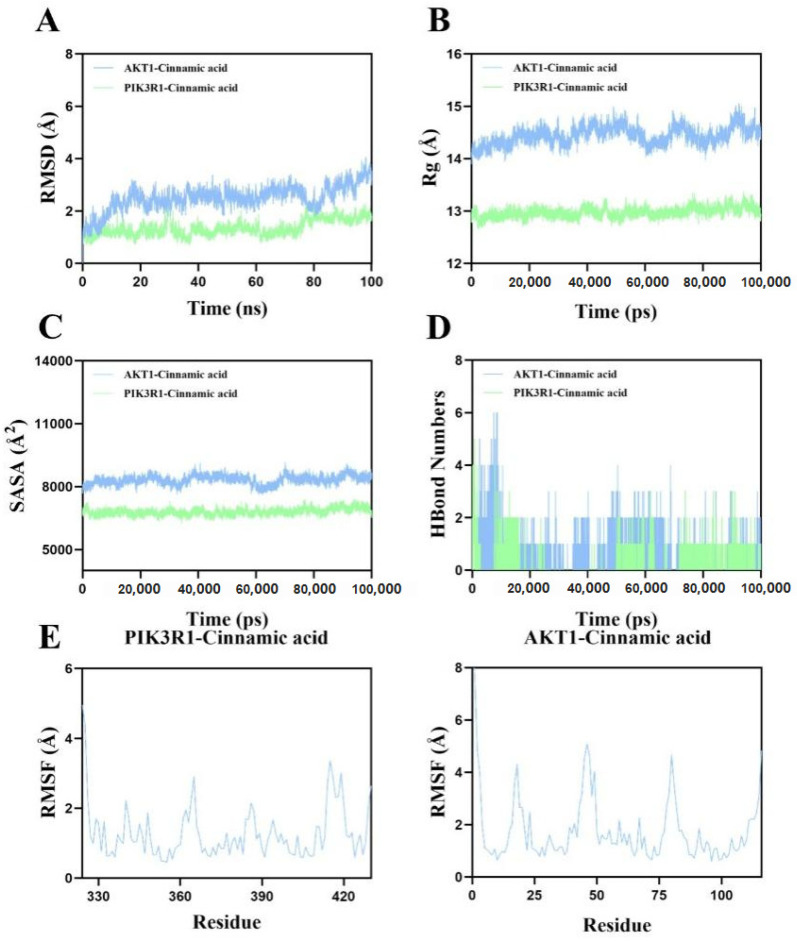
Molecular dynamics analysis of cinnamic acid complexes. (**A**) Backbone RMSD; (**B**) radius of gyration; (**C**) SASA; (**D**) intermolecular hydrogen bonds; and (**E**) per-residue RMSF for PIK3R1 (left) and AKT1 (right). Trajectories were recorded over 100 ns at 310 K and 1 bar.

To evaluate the time-dependent stability of the docked complexes, 100 ns all-atom MD simulations were carried out for AKT1–cinnamic acid and PIK3R1–cinnamic acid in explicit solvent (CHARMM36/CGenFF, TIP3P water; see Section 2.5 for details). The principal trajectory metrics are summarized in Figure 12. Panel A present the results of root mean square deviation (RMSD). Both complexes promptly reached equilibrium and fluctuated within narrow limits. The AKT1 complex stabilized between 20–80 ns with a mean RMSD of ~3.1 Å, while the PIK3R1 complex equilibrated after ~80 ns and remained around 1.8 Å. These low deviations indicate that cinnamic acid preserves a near-native protein backbone conformation throughout the simulation. Panel B present the results of the radius of gyration (Rg). Minor oscillations (≤0.5 Å) were observed for each complex, suggesting that ligand binding does not provoke global unfolding or compaction and that the overall tertiary architecture remains intact. A gradual convergence of solvent-accessible surface area (Figure 12C) implies that small local rearrangements around the binding pocket, rather than large-scale exposure/burial of surface residues, accommodate the ligand. Both complexes maintained an average of two intermolecular hydrogen bonds during the trajectory (0–6 for AKT1, 0–5 for PIK3R1; Figure 12D). Persistent H-bonding underpins the favorable docking poses identified in silico and contributes to complex stability. Panel E present the results of root mean square fluctuation (RMSF). Residue-wise flexibility remained low (predominantly <3 Å) for both proteins, with no evidence of allosteric destabilization distal to the binding site. Slightly elevated peaks correspond to solvent-exposed loop regions typical of native dynamics. Collectively, these data confirm that cinnamic acid forms stable, moderately rigid complexes with both PI3K regulatory subunit (PIK3R1) and AKT1. The sustained hydrogen bond network and minimal structural drift corroborate its favorable binding energies (−5.4 and −5.1 kcal mol^−1^, respectively) and lend further credence to the PI3K/AKT pathway as a principal pharmacological target of cinnamic acid in hepatocellular carcinoma.

## 3. Discussion

This study systematically reveals the multi-target mechanism of cinnamic acid against hepatocellular carcinoma through integrated machine learning prediction, network pharmacological analysis and in vitro experimental verification. The efficient screening ability of machine learning models provides new ideas for the activity prediction of natural products, and the combination of network pharmacological target prediction and molecular docking further narrows the scope of targets for experimental verification [25]. It is worth noting that the inhibitory effect of cinnamic acid on the PI3K/AKT signaling pathway is highly consistent in the calculation prediction and experimental verification: molecular docking shows its strong binding ability to PIK3R1 and AKT1 (binding energy < −5 kcal/mol), while Western blot confirmed that it significantly downregulates PI3K and p-AKT protein expression. This ‘prediction-verification’ closed-loop strategy not only improves the efficiency of natural product mechanism research, but also provides a methodological reference for the development of complex multi-target drugs.

The regulatory effect of cinnamic acid on the proliferation, migration and apoptosis of Hep3B cells may be closely related to its targeting of the PI3K/AKT pathway. The PI3K/AKT pathway is one of the core drivers of liver cancer progression, and its activation can promote tumor cell survival, angiogenesis and chemotherapy resistance [26]. This study found that cinnamic acid significantly reduces p-AKT levels by inhibiting key nodes of this pathway, thereby activating the mitochondrial apoptosis pathway (the Bax/Bcl-2 ratio is increased, and Caspase-3/8 expression is upregulated). This mechanism is complementary to the previously reported effect of cinnamic acid on the inhibition of the EGFR signal [27] in breast cancer and the regulation of the Wnt/β–catenin pathway [28] in colon cancer, suggesting that its anti-tumor effect has both broad-spectrum and pathway specificity. In addition, the inhibition of cinnamic acid on cell migration may be related to its regulation of MAPK signaling pathway or matrix metalloproteinase activity [29], though the specific mechanism still needs further exploration.

While our data confirm the multi-target nature of cinnamic acid, its full mechanism remains incompletely understood. Beyond the PI3K/AKT core, network pharmacology analysis uncovered 185 overlapping targets, including inflammatory regulators NFKB1 and RELA [30], suggesting that cinnamic acid may enhance its anti-liver cancer effect by inhibiting the tumor-associated inflammatory microenvironment. Heat shock protein 90α (HSP90AA1) in the core target is often highly expressed in liver cancer and is associated with adverse prognosis [31], and its strong binding to cinnamic acid (−5.7 kcal/mol) may provide new ways to overcome a drug resistant target. This raises the possibility that cinnamic acid also modulates the tumor-associated inflammatory micro-environment, thereby amplifying its anti-HCC activity. The breadth of these interactions underscores the therapeutic appeal of natural products that exert *multi-pathway* regulation, but it also highlights the need for more comprehensive mechanistic studies integrating transcriptomics, proteomics and in vivo validation.

In recent years, immune checkpoint inhibitors (e.g., anti-PD-1/PD-L1 antibodies) and targeted therapies (e.g., sorafenib, lenvatinib) have substantially changed the treatment landscape of advanced HCC [32]. However, issues such as drug resistance, high treatment costs, and adverse effects persist. Cinnamic acid, with its multi-target and low-toxicity profile, may provide a viable complementary or alternative approach to these strategies. By downregulating the PI3K/AKT pathway, a central node in HCC progression, cinnamic acid could potentially overcome or delay resistance mechanisms in patients who receive standard targeted agents or immunotherapies [33]. Future research could investigate the question of whether combining cinnamic acid with immune checkpoint inhibitors yields synergistic benefits, particularly by modulating the tumor immune microenvironment [34].

The limitations of this study are mainly reflected in the lack of in vivo experiments and the insufficient depth of mechanism research. Although in vitro experiments have confirmed the inhibitory effect of cinnamic acid on Hep3B cells, its efficacy, metabolic properties and potential toxicity in animal models still need to be verified [31]. In addition, the interaction between downstream effector molecules (such as mTOR, GSK-3β) and other pathways (such as MAPK, JAK/STAT) of the PI3K/AKT pathway has not been fully discussed [35]. In this study, we systematically elucidated the multi-target mechanism of cinnamic acid against hepatocellular carcinoma, focusing on the PI3K/AKT signaling pathway. Although our Western blot results demonstrate that cinnamic acid reduces total AKT and PI3K protein levels, we did not measure phosphorylated AKT (p-AKT), which is a direct readout of AKT’s enzymatic activity. This limitation precludes definitive conclusions regarding the extent of AKT inactivation by cinnamic acid. In future investigations, measuring the p-AKT/AKT ratio will be essential to fully clarify how cinnamic acid modulates PI3K/AKT pathway activity. Such additional data would strengthen the understanding of its underlying anti-tumor mechanisms and support potential translational applications. Future research can combine single-cell sequencing or spatial transcriptome technology to analyze the impact of cinnamic acid on immune cell infiltration and intercellular communication in the tumor microenvironment, and explore its synergistic effects with existing targeted drugs, such as sorafenib.

From the perspective of translational medicine, although the IC_50_ value of cinnamic acid (33.70 μM) is higher than that of some clinical chemotherapy drugs, its low toxicity (the safety of traditional applications has been verified) and multi-target characteristics mean that it has potential for combination medication. For example, cinnamic acid has shown low cytotoxicity in normal cells in reported studies, indicating a favorable safety profile. For instance, previous research has found that cinnamic acid significantly reduces the viability of cancer cells while having minimal effect on normal fibroblast cells, underscoring its selective toxicity toward tumor cells. This selective cytotoxicity suggests that cinnamic acid could be an effective anticancer agent, with relatively low side effects in normal tissues [36]. Moreover, the use of PI3K/AKT inhibitors may reduce the risk of drug resistance [37], or coordinate with immune checkpoint inhibitors to remodel the anti-tumor immune response [38]. In addition, derivatization modification based on the chemical structure of cinnamic acid, such as the introduction of hydrophilic groups or the development of nanodelivery systems [39], may further enhance its bioavailability and targeting.

In summary, this study systematically elucidates the molecular mechanisms by which cinnamic acid combats liver cancer through interdisciplinary strategies. It provides a theoretical basis for clinical translation and serves as an important reference for the contemporary research paradigm of natural products. Future research should validate its efficacy in in vivo models and explore its potential value in regulating the tumor microenvironment and immune response.

## 4. Methods

### 4.1. Construction of Anti-Tumor Prediction Model

This study used the PubChem, ChEMBL and RCSB databases [40,41,42] as data sources, small molecules with documented anti-tumor activity (IC_50_/EC_50_ ≤ 10 μM) were then screened as positive samples by searching keywords such as “Anti-neoplastic” and “Anti-tumor” (tag “1”), negative samples (label “0”) were randomly selected from 13 drugs (such as antiviral drugs) that have not been approved for antitumor indications to balance the data set [43]. The molecular characterization employs a 1024-bit Morgan fingerprint [44] and its topological characteristics are suitable for high-dimensional drug effect relationship modeling. The model training uses four algorithms: random forest (RF), support vector machine (SVM), gradient boost (GBoost) and logistic regression (LR). RF is suitable for high-dimensional features due to its strong noise resistance [45], SVM is good at handling nonlinear relationships [46], GBoost captures deep structure active associations through iterative optimization [47], and LR provides high interpretability probability output [48]. The above algorithm has been widely verified in virtual drug screening [49]. Though deep learning performs well in high-dimensional data processing (such as images and gene data) [50], its “black box” feature limits interpretability [51]; therefore, this study was not included. Data preprocessing completes feature extraction and numerical calculation through pandas and numpy. After StandardScaler standardizes the data, the training set and test set are divided in a respective 8:2 ratio and GridSearchCV optimizes hyperparameters [52,53]. Model performance is evaluated with accuracy, F1 score, AUC and sensitivity, and the visual classification tendency of the ROC curve and confusion matrix [54,55]. Finally, the Morgan fingerprint of cinnamic acid is input to the optimal model to predict activity, and 100 small molecules are randomly selected as external verification sets [44].

### 4.2. Screening and Mechanism Analysis of Network Pharmacological Targets

The database information and software information required for online pharmacology are shown in Table 4.

The database information and software information required for network pharmacology are shown in Table 4. All target names were standardized based on the UniProt database. For the collection of cinnamic acid targets, we collected its potential targets by integrating data from TCMSP (version 2.3), SuperPred (version 2.0), SwissTargetPrediction (version 2019-07-20), and CTD. After removing duplicates, target symbols were unified through the UniProt database. Collection of liver cancer targets was achieved using the keyword “Liver cancer,” with disease-related targets retrieved from DisGeNet (version 7.0), DrugBank (version 5.1.10), GeneCards, MalaCards, OMIM, and TTD. All retrieved targets were merged and duplicates were removed. Intersection and PPI network analysis was achieved by identifying the overlapping targets of cinnamic acid and liver cancer via Venny 2.1.0, yielding 185 intersection targets. These were uploaded to the STRING database (version 11.5), with species limited to “Homo sapiens.” We set the minimum required interaction score to 0.4 (“medium confidence”) and selected both “Experiments” and “Databases” as active interaction sources. Disconnected nodes were hidden to focus on the main network. The protein–protein interaction (PPI) network was then exported and visualized using Cytoscape (version 3.10.2). Core targets were screened through topological parameters within Cytoscape. Functional enrichment analysis was achieved via functional annotation and pathway enrichment, performed using the DAVID database (version 6.8). We used a threshold of *p* < 0.05 for significance in both gene ontology (GO) and KEGG pathway analyses. GO annotations included biological processes (BP), cellular components (CC), and molecular functions (MF). KEGG pathway analysis identified primary regulatory networks (e.g., PI3K/AKT, MAPK, apoptosis pathways). We then constructed a “component–pathway–target–disease” interactive network by integrating the top-ranked pathways (based on *p*-values) and their corresponding targets.

### 4.3. Molecular Docking Verification

To investigate the binding affinity between cinnamic acid and the core targets, we downloaded the crystal structures of AKT1 (PDB ID: 4EJN) and PIK3R1 (PDB ID: 3HHM) from the RCSB PDB database [65], All water molecules and irrelevant ligands were removed using PyMOL (version 2.5). The three-dimensional structure (SDF format) of cinnamic acid was obtained from the PubChem database [58], and then subjected to energy minimization in Chem3D (version 19.1), followed by conversion to PDB format. Docking was conducted using AutoDock Vina (version 1.2.3) [66] under default semi-flexible parameters. AutoDock Vina is an automated molecular docking program that predicts and optimizes binding modes between a protein and a small-molecule ligand by evaluating their interaction energies. It employs an empirical scoring function that accounts for van der Waals interactions, electrostatic (Coulombic) forces, hydrogen bonding, and hydrophobic effects; by minimizing this scoring function, Vina identifies the most stable binding pose for the ligand. In our protocol, a binding energy less than −5.0 kcal/mol was considered indicative of effective binding [67]. Finally, docking conformations were visualized using PyMOL to analyze hydrogen bonds and other interactions at the binding site.

### 4.4. Cell Experiments and Mechanism Verification

Human liver cancer cell line Hep3B (Institute of Biochemistry and Cell Biology, Chinese Academy of Sciences, Cat. No. SCSP-531, Shanghai, China) was cultured with 10% fetal bovine serum (FBS, Gibco 16000-044, Shanghai, China) and 1% penicillin–streptomycin (HyClone SV30010, Beijing, China) DMEM medium (Gibco 11965-092, Shanghai, China) was passed on in a 37 °C, 5% CO_2_ incubator (Thermo Fisher, Chinese branch offices, 51026280, Shanghai, China) [68]. Cinnamic acid (Sigma-Aldrich, Chinese branch offices C80857, Beijing, China) was dissolved in DMSO to prepare a 0–100 μM concentration gradient working solution (final concentration < 0.1%). The experimental group added the drug according to the concentration gradient, and the control group added an equal volume of DMSO.

Cell proliferation detection was performed by CCK8: Hep3B cells were inoculated with a density of 6 × 10^3^ per well in a 96-well plate (Corning, Chinese branch offices, 3599, Shanghai, China), and the drug-containing culture medium was replaced 24 h later, with 6 compound wells set in each group. After 24, 48 and 72 h, CCK8 reagent (Dojindo CK04) was added to incubate for 2 h. The microplate reader (BioTek Synergy H1, Beijing, China) measured the absorbance of 450 nm and calculated the survival rate [69]. The formula for calculating cell survival is as follows: (experimental group OD − blank group OD)/(control group OD − blank group OD) × 100%. In cell morphology observation, Hep3B cells were seeded in 6-well plates at 3 × 10^5^ per well (Corning, 3516), with different concentrations of cinnamic acid added for 24 h, and with inverted microscopes being used (Olympus, Chinese branch offices, IX73, Shanghai, China) at 24 and 48 h. We observed the cell morphology and photographed and recorded it. Images were captured at a magnification of 200× (objective lens), and a scale bar of 500 µm was embedded in each micrograph to indicate the actual size.

Cell migration experiments were subjected to scratching method, and Hep3B cells were seeded on 6-well plates at 3 × 10^5^ per well. After culturing until the cell fusion degree reached more than 90%, a straight line was drawn on the monolayer cells with a 200 μL sterile gun head. We then scratched and PBS washed the cultures to remove shed cells. We added different concentrations of cinnamic acid, continued to culture and took photos with an inverted microscope at 0, 12, 24 and 48 h [70]. Images were captured at a magnification of 200× (objective lens), and a scale bar of 500 µm was embedded in each micrograph to indicate the actual size. We used ImageJ software (version imageJ-fiji) to calculate the scratch healing rate (%) = (initial scratch width − scratch width after treatment)/initial scratch width × 100%. No formal statistical tests were performed due to the lack of replicate samples in each group.

Hep3B cells were seeded in 6-well plates at 3 × 10^5^ per well. After 48 h of drug treatment, the drug was digested and collected with trypsin (Gibco, 25200-072), washed twice with PBS, and resuspended in 1× Binding Buffer (BD Biosciences, Chinese branch offices, 556547, Beijing, China). We added Annexin V-FITC (5 μL) and PI (5 μL) (BD Biosciences, 556547) and incubated for 15 min away from light. Apoptosis rate was detected using flow cytometry (BD FACSCanto II). FlowJo10.8.1 software was used to analyze the proportion of early (Annexin V^+^/PI^−^) and late apoptosis (Annexin V^+^/PI^+^) cells [70].

Western blot was used to detect protein expression. After 48 h of drug treatment, total protein was extracted with RIPA lysate (Beyotime, China, P0013B, Shanghai, China), and concentration was determined by the BCA method (Thermo Fisher, 23227). We took 30 μg of protein for SDS-PAGE electrophoresis (Bio-Rad, Chinese branch offices, 4568083, Shanghai, China), transferred the membrane to a PVDF membrane (Millipore, Chinese branch offices, IPVH00010), blocked 5% skim milk for 1 h, added primary antibody—PI3K (CST, Chinese branch offices, 4257, 1:1000, Shanghai, China), AKT (CST, 40D4, 1:2000, Shanghai, China), Bcl-2 (Abcam, Chinese branch offices, ab32124, 1:1000, Shanghai, China), Bax (Abcam, ab32503, 1:1000, Shanghai, China), β-actin (Proteintech, Chinese branch offices, 66009-1-Ig, 1:5000, Wuhan, China)—and incubated overnight at 4 °C. After washing TBST and HRP-labeled secondary antibody (Proteintech, SA00001-1, 1:5000, Wuhan, China), we incubated for 1 h at room temperature for ECL development (Advansta, K-12045-D50, Beijing, China) and analyzed the grayscale value by ImageJ [71].

### 4.5. Molecular Dynamics (MD) Simulation

All-atom MD simulations were carried out with GROMACS 2022 for the two docked complexes (AKT1–cinnamic acid and PIK3R1–cinnamic acid). Regarding system preparation, protein topologies were generated with the CHARMM36m forcefield, and ligand parameters were obtained from CGenFF v4.6 through the CHARMM-GUI/ParamChem interface. Each complex was centered in a dodecahedral box leaving ≥1.0 nm between any solute atom and the box edge, then solvated with TIP3P water. Counter-ions, together with 0.15 M NaCl, were added using gmx genion to neutralize the system. Energy minimization and equilibration were achieved as follows: after 3000 steps of steepest-descent followed by 2000 steps of conjugate-gradient minimization, a two-stage equilibration was performed via (i) 500 ps NVT heating from 0 K to 310 K with heavy-atom restraints (k = 1000 kJ mol^−1^ nm^−2^) using the V-rescale thermostat and (ii) 1 ns NPT equilibration at 310 K and 1 bar with the Parrinello–Rahman barostat. Production run was undertaken via collection of un-restrained production trajectories of 100 ns in the NPT ensemble with a 2 fs integration step (leap-frog algorithm, LINCS constraints on all bonds). PME handled long-range electrostatics (real-space cut-off 1.0 nm), and Lennard–Jones interactions used a 1.0 nm cut-off with force-switch smoothing. Coordinates were saved every 10 ps for analysis; Trajectory analyses were achieved as follows: backbone RMSD (gmx rms), per-residue RMSF (gmx rmsf), radius of gyration (gmx gyrate), solvent-accessible surface area (gmx sasa), and intermolecular hydrogen bonds (gmx hbond) were computed over the full 100 ns trajectory.

### 4.6. Statistical Analysis

Experimental data are expressed as mean ± standard deviation (Mean ± SD) and graphs are drawn using GraphPad Prism 9.0 software. One-way analysis of variance (ANOVA) was used for the inter-group comparison, and the Student’s *t*-test was used for the difference analysis between the two groups. The difference was statistically significant in *p* < 0.05.

## 5. Conclusions

This study systematically reveals the molecular mechanism of cinnamic acid anti-hepatocellular carcinoma (HCC) through integrated machine learning prediction, network pharmacology and in vitro experimental verification. The random forest model predicts that cinnamic acid has significant anti-tumor activity (probability = 0.69), which inhibits Hep3B cell proliferation (IC_50_ = 33.70 μM) and migration by targeting the PI3K/AKT signaling pathway (core targets PIK3R1, AKT1). The healing rate of the μM group decreased to 10.8%) and apoptosis was induced (the Bcl-2/Bax ratio was significantly reduced). Molecular docking confirmed that the binding energy of cinnamic acid with the key target PIK3R1 was −5.4 kcal/mol, while with the key target AKT1 it was −5.1 kcal/mol, indicating high affinity (binding energy < −5 kcal/mol).

Cinnamic acid demonstrates promising application prospects in the field of anti-hepatocellular carcinoma. Future research could explore several key directions: Firstly, in the further elucidation of its molecular mechanisms, particularly in regulating key signaling pathways such as PI3K/AKT and NF-κB; secondly, in the development of novel cinnamic acid derivatives or nano-delivery systems that could enhance its bioavailability and targeting efficacy while minimizing side effects. Additionally, investigating the combined application of cinnamic acid with existing chemotherapy drugs, such as sorafenib, may improve therapeutic efficacy and overcome drug resistance through synergistic effects. Finally, integrating artificial intelligence to screen for highly effective and less toxic cinnamic acid analogs, followed by conducting preclinical and clinical trials, would facilitate its translation into clinical practice. These studies are expected to provide new strategies and drug candidates for the treatment of hepatocellular carcinoma.

## Figures and Tables

**Table 1:** Performance of the different anti-tumor prediction models.

Model	Accuracy	F1 Score	AUC	Sensitivity
RF	0.80	0.80	0.86	0.76
GBoost	0.74	0.74	0.82	0.71
LR	0.72	0.72	0.77	0.69
SVM	0.78	0.78	0.85	0.73

**Table 2:** Random forest model predicted the top 20 molecules.

Model	Probability	Compound	Model	Probability	Compound
RF	0.80	Random 1	RF	0.65	Random 10
RF	0.76	Random 2	RF	0.65	Random 11
RF	0.70	Random 3	RF	0.65	Random 12
RF	0.69	Cinnamic acid	RF	0.64	Random 13
RF	0.69	Random 4	RF	0.63	Random 14
RF	0.68	Random 5	RF	0.62	Random 15
RF	0.68	Random 6	RF	0.62	Random 16
RF	0.66	Random 7	RF	0.62	Random 17
RF	0.65	Random 8	RF	0.62	Random 18
RF	0.65	Random 9	RF	0.62	Random 19

**Table 3:** Receptor information and binding energy.

Target Protein	Binding Energy (kcal/mol)	Target Protein	Binding Energy (kcal/mol)
RXRA	−7.1	PIK3R1	−5.4
CHRM1	−6.7	NFKB1	−5.4
PDGFRA	−6.3	MAPK1	−5.4
BCL2	−6.0	FLT1	−5.2
GSK3B	−5.9	NOS3	−5.2
TLR4	−5.9	ITGB7	−5.2
RELA	−5.8	MCL1	−5.2
HSP90AA1	−5.7	IL6	−5.1
ERBB2	−5.6	AKT1	−5.1
EGFR	−5.5		

**Table 4:** Database information.

Database	Website
DAVID [56]	https://david.ncifcrf.gov/ accessed on 24 January 2025
GeneCards [57]	https://www.genecards.org/ accessed on 24 January 2025
PubChem [40]	https://pubchem.ncbi.nlm.nih.gov/ accessed on 24 January 2025
STRING [58]	https://cn.string-db.org/ accessed on 24 January 2025
Superpred [59]	https://prediction.charite.de/index.php accessed on 24 January 2025
TCMSP [60]	https://www.91tcmsp.com/ accessed on 24 January 2025
SwissTargetPrediction [61]	http://swisstargetprediction.ch/ accessed on 24 January 2025
CTD [62]	https://ctdbase.org/ accessed on 25 January 2025
UniPort [63]	https://www.uniprot.org/ accessed on 26 January 2025
Venny 2.1.0	https://bioinfogp.cnb.csic.es/tools/venny/ accessed on 24 January 2025
Weisheng Xin	https://www.bioinformatics.com.cn/ accessed on 26 January 2025
Cytoscape 3.10.2 [64]	http://www.cytoscape.org accessed on 27 January 2025
PDB	https://www.rcsb.org/ accessed on 22 January 2025
GenBank	https://www.ncbi.nlm.nih.gov/ accessed on 23 January 2025

## Data Availability

Data is contained within the article.
